# Hybrid Wetting Surface with Plasmonic Alloy Nanocomposites for Sensitive SERS Detection

**DOI:** 10.3390/molecules28052190

**Published:** 2023-02-27

**Authors:** Shanjiang Wang, Dan Su, Huanli Zhou, Xiaohan Jiang, Xiaoyang Zhang, Tong Zhang

**Affiliations:** 1 School of Materials Science and Engineering, Nanjing Tech University, Nanjing 210009, China; 2 Joint International Research Laboratory of Information Display and Visualization, School of Electronic Science and Engineering, Southeast University, Nanjing 210096, China

**Keywords:** hybrid wetting surface, SERS, hot spots, condensation effects, sensor-based applications

## Abstract

In this paper, a hybrid wetting surface (HWS) with Au/Ag alloy nanocomposites was proposed for rapid, cost-effective, stable and sensitive SERS application. This surface was fabricated in a large area by facile electrospinning, plasma etching and photomask-assisted sputtering processes. The high-density ‘hot spots’ and rough surface from plasmonic alloy nanocomposites promoted the significant enhancement of the electromagnetic field. Meanwhile, the condensation effects induced by HWS further improved the density of target analytes at the SERS active area. Thus, the SERS signals increased ~4 orders of magnitude compared to the normal SERS substrate. In addition, the reproducibility, uniformity, as well as thermal performance of HWS were also examined by comparative experiments, indicating their high reliability, portability and practicability for on-site tests. The efficient results suggested that this smart surface had great potential to evolve as a platform for advanced sensor-based applications.

## 1. Introduction

Facile, rapid, reproducible, as well as low-cost trace analyte detection has attracted much attention in the field of diagnosis, food safety analysis, molecule detection, and so on [1,2,3,4]. Among other optical techniques, for example, plasmonic sensors with unpolarized light input and simple optics-based spectral signal systems [5], fluorescence-based sensors with low background and simple operation [6], surface-enhanced Raman scattering spectroscopy (SERS) is deemed to be effective access due to its high sensitivity, which mainly depends on the enhanced local electromagnetic field near the detection area and then amplifies Raman signals of absorbed target molecules [7,8,9]. Therefore, for a typical sensitive SERS process, two issues must be taken into consideration, one is how to design and prepare SERS substrates with high sensitivity and reproducibility in a convenient way, and another is the way to achieve high condensation and uniform adsorption of target molecules around the detecting area. Recently, great developments have been made in achieving sensitive and broad-spectrum SERS signals based on methods such as top-down lithography [10,11], deposition techniques [12] or template-assisted routes [13,14]. However, most of these methods rely on sophisticated equipment or complicated preparation processes, which naturally increase costs and inconvenience for rapid and large-scale operations. Furthermore, dipping the SERS substrate into a target molecule solution is a common way to realize uniform adsorption. Still, such processes need much more time (several hours) due to the low diffuse rate (ca. 60 h/cm) [15,16]. In addition, substrate waste cannot be avoided because of the limited area relying on the spot size of the laser source.

Plasmonic nanostructures have been extensively used for sensitive SERS substrates, benefiting from their morphology-related enhancement of local electromagnetic fields [17,18,19]. The formed nanoscale gaps among aggregative nanoparticles, known as the “hot spots” [20,21], can dramatically enhance the local electromagnetic field. Thus, the Raman signals from trapped molecules inside or close enough gaps can be strongly enhanced. Such a result could be ascribed to the collective surface plasmon resonances (SPRs) effects with interparticle plasmon coupling. In previous works, spherical metallic nanoparticles (NPs) are reported as suitable candidates due to their superior physicochemical stability and reproducibility on a large scale [22,23,24]. However, nanoparticles with sole elements showed limited enhancement factors due to restricted numbers of “hot spots” and a mismatch between the plasmon resonance wavelength to the excitation laser wavelength. In such cases, sensitive and broad-spectrum SERS signal platforms by hot spots have great potential for practical applications, and they have also been reported in pioneering works, including an AuNP-rGO-silicon wafer hybrid detection platform [25] with tunable coupling distance, CRISPR-Cas12a protein assisted core-satellite nanocluster [26] and multilayered mesoporous gold nanoarchitecture [27]. Hence, the design of multiple plasmonic hot spot generation by nano-gap-rich metal nanostructures is a growing interest for improving SERS activities (i.e., the substrates made of aggregations of Au/Ag).

**Figure 1:**
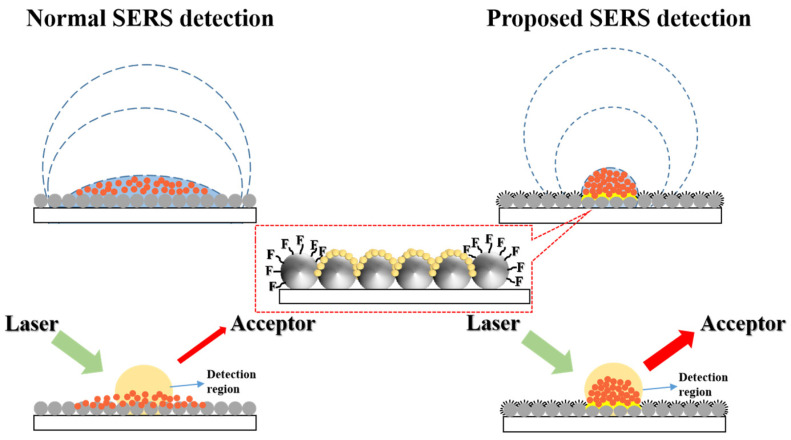
Schematic of normal SERS detection (**Left**) and proposed SERS detection (**Right**). For normal SERS detection, the droplet spreads over the substrate by capillarity and then continuously and completely evaporates; the limited detection sensitivity could be attributed to the restricted detection region of instruments (e.g., spot size of laser) versus the sparse distribution of target analytes. In contrast, SERS substrate with HWS could highly concentrate analytes within the detection region due to high hydrodynamic-flow resistance and then increase the limit of detection. Inset (red dashed box) showed HWS acquired by sputtering Au NPs (hydrophilic) and modifying—F groups (hydrophobic), respectively.

On the other hand, to overcome the limit for trace detection, hybrid wetting surfaces (HWS) with tunable hydrophobic–hydrophilic wetting properties have begun to attract attention due to their ability to enrich target analytes into a fixed small area [28,29,30]. As shown in Figure 1 (Left), the droplet can spread over the substrate by capillarity and then continuously and completely evaporates; in such cases, the target molecules are difficult to concentrate, which decreases the detection sensitivity (especially for trace analytes) and increases the requirements for instruments. In contrast, for HWS (Figure 1 Right), the deposited droplet is restrained and pinned within the superhydrophilic area because of the high hydrodynamic flow resistance brought by the surrounding superhydrophobic structure [31]. As a result, the droplet containing target molecules could evaporate without flowing outward, and thus the homogeneous distribution of molecules is realized. In such cases, substrate wastage could be avoided. Meanwhile, the sensitivity of the SERS substrate increases remarkably. In addition, it is also suitable for rapid positioning detection without the help of large confocal optical microscopy. In the previous works, the reported HWS mainly included randomly arranged nanostructures [32] and controllable periodic structures [33]; the frangibility and ease in peeling off are difficult to solve for the former, but the sophisticated process and the high cost from the latter are also obstacles for further application. Hence, the facile way for well-designed plasmonic structures is challenging for HWS. To further broaden the SERS application, it is also important to explore scalable, reproducible approaches as well as cost-effective preparation routes to achieve SERS substrates with ultrahigh SERS activities.

In this study, we demonstrate a simple way to fabricate HWS with Ag-Au/Ag nanocomposites through electrospinning, sputter coating and surface modification processes. As a result, Au/Ag alloy nanocomposites show extremely rough surfaces and strong ‘hot spots’ effects compared to homogeneous nanoparticles. In addition, benefiting from HWS, the enrichment and highly sensitive detection of diverse target analytes at the SERS-active area are also achieved with a reproducible, stable, rapid as well as low-cost pathway, which exhibits the potential for practical application, such as the development of various advanced chemical and biological sensors.

## 2. Results and Discussion

**Figure 2:**
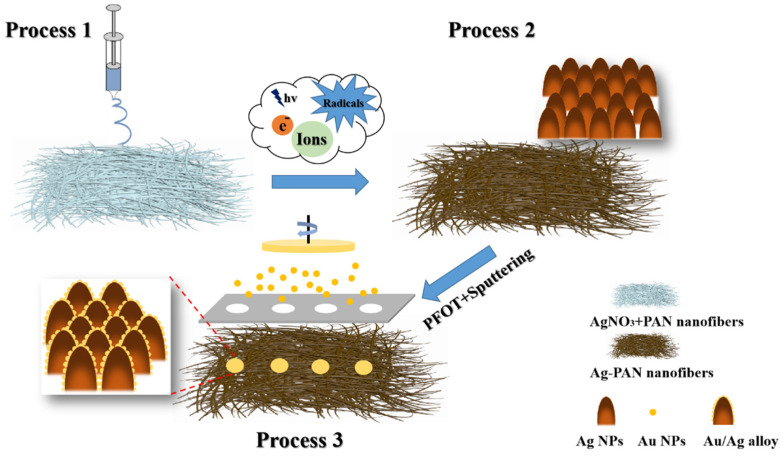
Fabrication of hydrophilic Au/Ag alloy nanocomposites on the hydrophobic Ag-PAN substrate. Process 1: electrospinning porous AgNO_3_-PAN nanofibers substrate. Process 2: Plasma etching process, crystalline Ag NPs were generated after the interaction between metal salts and activated species from plasma groups. Inset showed morphology of Ag NPs on the surface of nanofibers. Process 3: Selective sputtering Au NPs by using homemade masks. In such cases, -F groups were peeled off and then formed Au/Ag alloy nanocomposites, as shown in the inset of Process 3.

Based on the basic principles of HWS described above, the details for fabrication are illustrated in Figure 2. Firstly, the hydrophobic surface was prepared by electrospinning the AgNO_3_ and polyacrylonitrile (PAN) mixture, followed by the plasma etching process and hydrophobic modification through 1H, 1H, 2H, 2H-perfluorodecyltrimethoxysilane (PFOT). The crystalline metal NPs were generated after interacting with metal salts and the activated species from plasma groups. The high affinity between metal and -SH made it hydrophobic due to the bareness of -F groups. Next, the homemade photomask with designed hole sizes was used for preparing the hydrophilic surface (SERS active area) by combining magnetron sputtering routes. In such cases, the bombard from charged ions to the bare surface with -F groups turned hydrophobic to hydrophilic. Meanwhile, the Au atoms from the target were also peeled off and then deposited on the bare surface to form hydrophilic Au/Ag alloy nanocomposites.

**Figure 3:**
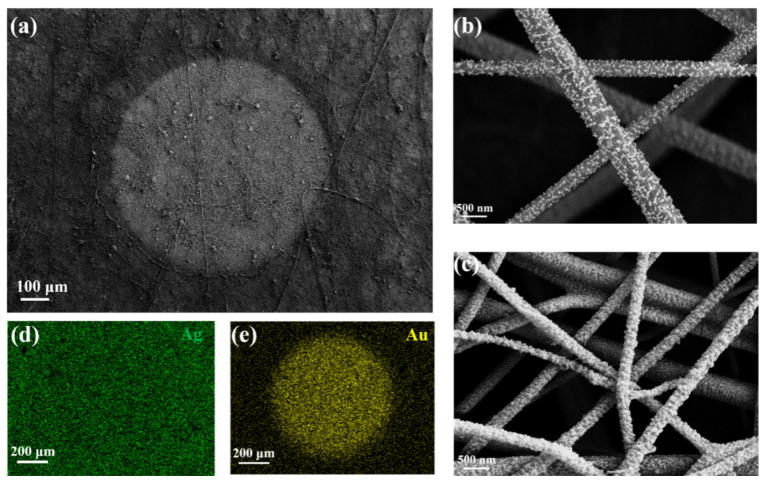
(**a**) SEM image of HWS. (**b**) The enlarged SEM images of hydrophobic Ag-PAN and (**c**) hydrophilic Au/Ag alloy nanocomposites, respectively. (**d**) The corresponding spatial element distribution of hydrophobic area and (**e**) hydrophilic SERS active area.

Subsequently, the morphology of the proposed HWS was described by SEM images and element analysis. As shown in Figure 3a, the bright white circle at the center was Ag/Au alloy nanocomposites, and the remaining area was undoubtedly Ag-PAN. The enlarged images in Figure 3b,c shows the matched results. There was a sparse surface distribution of Ag NPs on the fibers after the plasma etching process. However, the density and roughness significantly increased after Au NPs deposition mediated by the sputtering route (Figure 3c); the difference was also observed in reflectance spectrum results (Figure S1, Supplementary Materials). Moreover, the spatial element distribution in Figure 3d,e and Figure S2 confirmed that Au nanoparticles were deposited uniformly on the surface.

**Figure 4:**
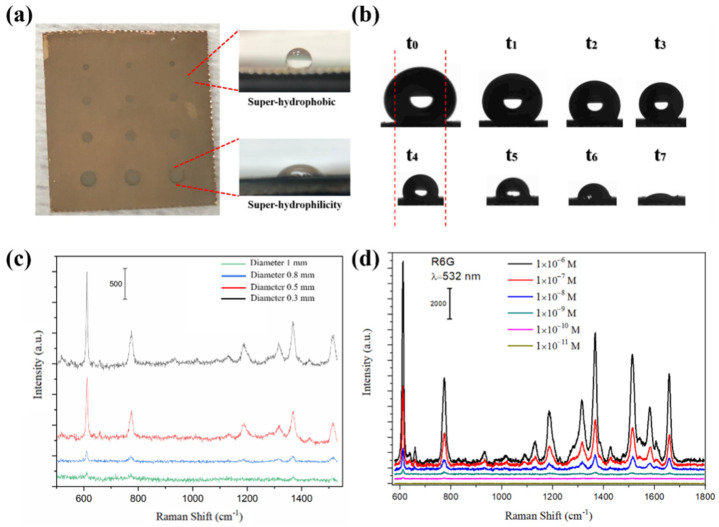
(**a**) Photographs of HWS, where the dark grey area showed hydrophilic properties with a contact angle of 21 ± 2° and the brown area showed hydrophobic properties with a contact angle of 151 ± 3°. (**b**) The evaporating evolution process of a 20 μL droplet containing 10^−7^ M R6G under room temperature. (**c**) The dependence of SERS signals intensities on the size of the SERS active area. (**d**) SERS spectra of different concentrations of R6G (1 × 10^−6^ to 1 × 10^−11^ M) on HWS.

To evaluate the HWS, the hydrophilicity/hydrophobicity properties were examined by measuring the contact angle of a water droplet on the different areas (see Figure 4a). The Ag-PAN with brown color was first modified by low-energy fluorosilane, which made the whole surface show hydrophobic properties with a contact angle of 151 ± 3°. Then, Au/Ag alloy patterns with tunable diameters were prepared by selective sputtering with the help of a homemade mask, which showed dark grey color with hydrophilic properties (a contact angle of 21 ± 2°). Figure 4b and Figure S3 showed the evaporating evolution process of a 20 μL droplet containing 10^−7^ M Rhodamine 6G (R6G) under room temperature. The spherical shape droplet showed hydrophobic properties at the stages t_0_ to t_3_, which pinned and kept on shrinking its contact line until totally confined in the hydrophilic area (t_4_); after that, the droplet collapsed at the hydrophilic area and then completely evaporated. In such cases, the target analytes were concentrated in the area of solid–liquid contact. They showed homogeneous distribution due to the inhibiting effect of the evaporation-driven advective flow brought by the high hydrodynamic flow resistance [34,35].

To examine the remarkable enhancement of the SERS activities for HWS, comparative experiments were conducted by measuring SERS spectra of normal SERS substrate with Au/Ag alloy-PAN and proposed HWS. Firstly, we studied the relationship between SERS signals and the size of SERS active areas. As shown in Figure 4c, the SERS intensity increased dramatically with the decrease in the diameter of the SERS active area due to the condensation of analyte molecules. Both SERS active areas with diameters of 0.5 mm and 0.3 mm showed obvious SERS signals; optimized in this work was the SERS active area with a 0.5 mm diameter when considering the water trapping capacity and repaid positioning as cost-effective. Under such circumstances, compared with the result of normal SERS substrate in Figures S4–S6, HWS showed stronger and clearer peaks with the detection sensitivities down to 10^−11^ M (Figure 4d), which was ~4 folds higher than that of normal SERS substrate, confirming that both ‘hot spots’ effects mediated by density Au/Ag alloy nanocomposites and condensation effect contributed to such activities enhancement.

**Figure 5:**
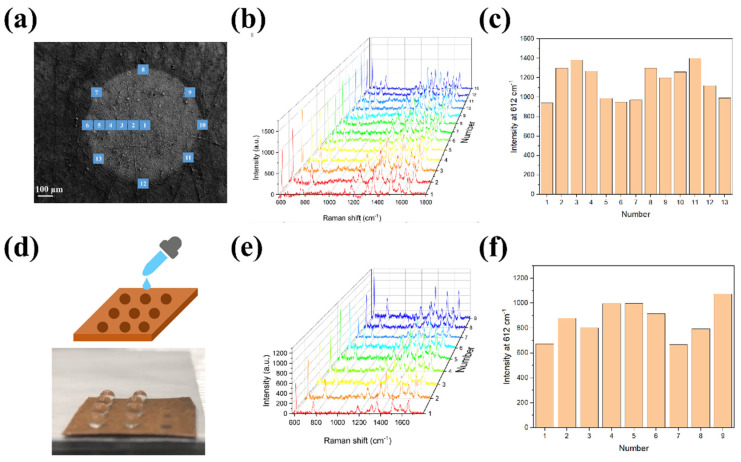
(**a**) SEM image of SERS active area with 13 marked spots. (**b**) The corresponding SERS spectra of R6G from 13 spots (**c**) and the histogram for the peak intensity at 612 cm^−1^. (**d**) Photograph of a SERS chip containing 9 SERS active areas by dropping 20 μL of R6G. (**e**) The corresponding SERS spectra of R6G from 9 different SERS active areas (**f**) and the histogram for the peak intensity at 612 cm^−1^.

After evaluating the superior performance of HWS for trace molecules detection, we investigated the uniformity and reproducibility of the SERS signal using this unique surface. As shown in the SEM image of Figure 5a, the SERS signals were measured by marking 13 spots on a single SERS active area, and the corresponding results are depicted in Figure 5b,c (the concentration of R6G was 1 × 10^−9^ M). Relative standard deviation (RSD) of SERS intensities from 13 different spots was calculated to be about 16.2% (Figure 5c). It could be observed that the SERS signals from the border showed a similar order of magnitude compared with that for the inner ones, indicating good uniformity after the condensation process. In addition, the intensity variations among different detection areas (Figure 5d) were tested using a portable Raman analyzer to examine the reproducibility (the concentration of R6G was 1 × 10^−9^ M). The results in Figure 5e,f demonstrated that the proposed SERS substrates possessed excellent reproducibility due to relatively consistent signal intensities. Corresponding RSD of SERS intensity from Figure 5f was calculated to be about 15.9%.

**Figure 6:**
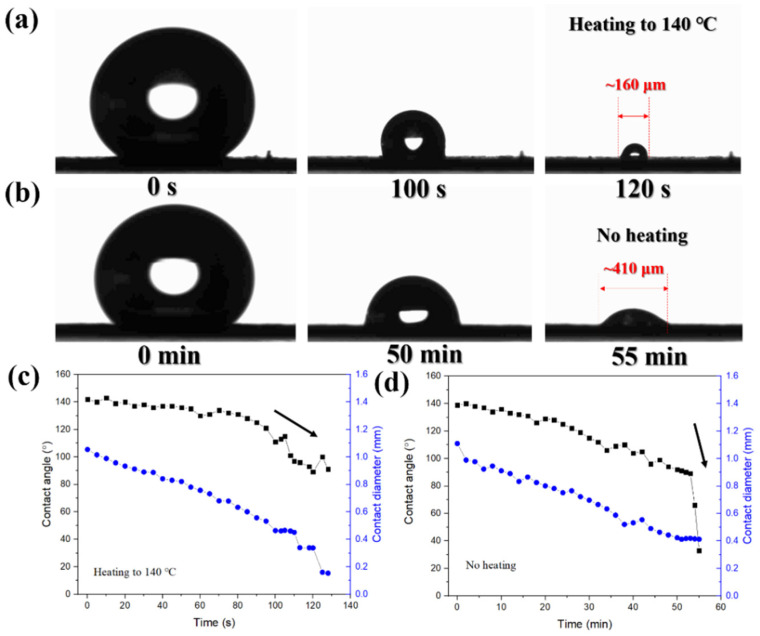
(**a**) The morphology evolution of the droplet during the concentration process on the HWS-based SERS substrate with a diameter of 500 μm and heated to 140 °C. (**b**) The morphology evolution of the droplet on the HWS-based SERS substrate with a diameter of 500 μm under no heating conditions. The evolution of contact angle and contact diameter over time during concentration in the heating process (**c**) and the non-heating process (**d**).

The thermal performance of the HWS substrate was then evaluated. Figure S7 shows negligible changes in SERS signals after treating the high temperature around ~200 °C within 15 min, suggesting the good heat endurance of HWS. We also made comparative experiments to investigate the morphology evolution of a droplet with or without the heating process. As presented in Figure 6a, under heating conditions at 140 °C, the shape of the droplet remained spherical, and the final contact area was 0.02 mm^2^ at ~120 s. However, the area decreased by ~6.6 folds, and the evaporation rate increased by ~27.5 folds compared to unheated ones, where the final contact area was 0.132 mm^2^ at ~55 min (Figure 6b). The corresponding changes in the contact angle and contact diameter with time under heating and non-heating processes are illustrated in Figure 6c,d, respectively. For both processes, the contact line of the droplet was continuously reduced until the droplet completely contracted into the hydrophilic area and then collapsed with a dramatic decrease in contact angle until the droplet dried. However, the contact angle of the droplet remained at a high value (above 90°) during the whole heating process (Figure 6c), and the spherical state was kept until it completely evaporated. The stable spherical state could be attributed to the formation of a water vapor film between the droplet and the substrate. Thus, the droplet could not collapse easily because of the wet transition compared with the non-heating process (Figure 6d) [26,30]. Therefore, such superior heating stability and unique concentration phenomenon could further achieve the condensation of target analytes at the hydrophilic area with the help of HWS. Meanwhile, the heating could also accelerate droplet evaporation and shorten the operation time, as expected, especially for detecting molecules with good thermal stability.

In conclusion, Au/Ag alloy ‘hot spots’ combined with HWS were fabricated by facile and low-cost multi steps including convenient electrospinning, plasma etching, surface modification and photomask-induced sputtering processes. The multiple plasmonic ‘hot spot’ generated by nano-gap-rich metal alloy nanocomposites guaranteed high sensitivity and uniformity of SERS signals. Moreover, as expected, the HWS property concentrated the target analytes and ensured homogeneous distribution at the SERS-active area. As a result, the SERS signals increased by ~4 orders of magnitude compared to the normal SERS substrate. Furthermore, the studies of reproducibility, uniformity as well as thermal stability demonstrated that such HWS-based SERS substrate also made it easily accessible to end-users without complicated fabrication steps for on-site detection in practical applications.

## 3. Experimental Section

Reagents: AgNO_3_ (99.9999% trace metals basis) and PAN (Mw = 150,000) powder were purchased from Sigma-Aldrich (St. Louis, MO, USA). N, N-Dimethylformamide (DMF, 99.5%) and 1H, 1H, 2H, 2H-perfluorodecyltrimethoxysilane (PFOTS, 97%) were obtained from Aladdin. Degassing distilled water (18.2 MΩ·cm) was used in all experiments. All the chemicals were analytical grade and used without further purification.

Fabrication Process: The AgNO_3_/PAN nanofibers substrate was prepared by dissolving 0.25 g AgNO_3_ powder in 10 g 10 wt% PAN/DMF mixture, continuously stirring in the dark for 12 h, followed by electrospinning for 4 h with a voltage of 15 kV, a spinning distance of 25 cm, a rotation drum at a speed of 20 rpm and a feeding rate of 1.0 mL/h with a 21-gauge needle tip. Then, the acquired sample was collected and treated with argon plasma (50 W, 100 mTorr, Harrick, Pleasantville, NY, USA) for 10 min. The hydrophobic surface was obtained by immersing Ag-PAN nanofibers substrate into PFOTs hexane solution (25 mM) for 18 h without disturbance; after that, the substrate was carefully removed and washed with hexane thoroughly to remove excess PFOTs. For preparing HWS, the resulting hydrophobic Ag-PAN nanofibers substrate was fixed with a homemade photomask with hole diameters of 1 mm, 0.8 mm, 0.5 mm and 0.3 mm. Then they were sputtered with Au NPs in a desk-based magnetron sputtering machine under 60 s to realize HWS.

Characterizations: The morphologies and elements analysis of HWS were characterized by scanning electron microscopy (SEM, Zeiss Sigma 300, Cologne, Germany). The hydrophilicity/hydrophobicity was evaluated using a contact angle measurement system (JY-82B Kruss DSA, Hamburg, Germany). Optical absorption was recorded by ultraviolet-visible-near-infrared spectrophotometer (PE lambda 750, Freehold, NJ, USA). Structure information was investigated by X-ray diffraction spectrum (XRD, Thermo Kalpha, Urbana, IL, USA). Raman scattering in Figure 5a,d was performed on a Raman microscope (LabRam HR Evolution, Villeneuve d’Ascq, France) equipped with a 532 nm laser as the excitation source at room temperature and a portable Raman analyzer, respectively.

## Data Availability

Not applicable.

## Supplementary Materials

The following supporting information can be downloaded at https://www.mdpi.com/article/10.3390/molecules28052190/s1, Figure S1: The reflectance spectrum of Ag-PAN and Au/Ag-PAN by using bright-field microscope; Figure S2: The element contents of Au and Ag within the SERS active area; Figure S3: The dependence of contact angle on the evaporation time; Figure S4: (a) SERS spectra of different concentrations (1 × 10^−5^ to 1 × 10^−8^ M) of R6G on normal SERS substrate with Au/Ag alloy-PAN, the detection limit of R6G on such substrate was measured to be 10^−8^ M. (b) the corresponding log-log plot of the average intensity of SERS signals at 612 cm^−1^ and R6G concentration. Figure S5: SERS intensity at 612 cm^−1^ of HWS (orange) and normal SERS substrate (green); Figure S6: SERS signals of R6G molecules (1 × 10^−1^ M) on the pure PAN substrate; Figure S7: The evolution of SERS signals before and after heating process (200 °C for 15 min). Table S1: The corresponding statistics from Figure S1.
