# Supplementary material for: Hybrid Wetting Surface with Plasmonic Alloy Nanocomposites for Sensitive SERS Detection
Source: Molecules. 2023 Feb 27;28(5):2190. doi: 10.3390/molecules28052190 (PMC10004610; doi:10.3390/molecules28052190)
Supplement: Supplementary file 1 [file molecules-28-02190-s001.zip › molecules-2229843-supplementary.pdf]

Hybrid Wetting Surface with Plasmonic Alloy Nanocomposites for Sensitive SERS Detection

Shan-Jiang Wang[1,2], Dan Su[2], Huan-Li Zhou[2], Xiao-Yang Zhang[2], Tong Zhang[2*]

[1]Jiangsu Collaborative Innovation Center for Advanced Inorganic Function Composites, School of Materials Science and Engineering, Nanjing Tech University, Nanjing 210009, People's Republic of China.

[2]Joint International Research Laboratory of Information Display and Visualization, School of Electronic Science and Engineering, Southeast University, Nanjing, 210096, People's Republic of China.

* Correspondence: tzhang@seu.edu.cn;

Define of hydrophobicity and hydrophilicity:

The wetting properties (hydrophobicity or hydrophilicity) could be determined by measuring contact angle (θ) between droplet and surface,

Super-hydrophobicity: contact angle (θ) > 150°, sliding angle (a) < 10°.

Hydrophobicity: 90° <contact angle (θ) < 150°

Hydrophilicity: contact angle (θ) < 90°

Super-hydrophilicity: contact angle (θ) < 5°

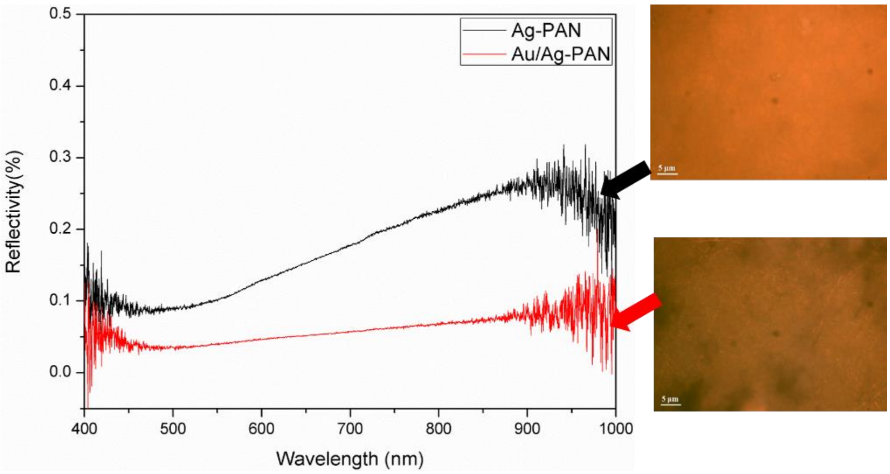

Figure S1. The reflectance spectrum of Ag-PAN and Au/Ag-PAN by using bright-field microscope.

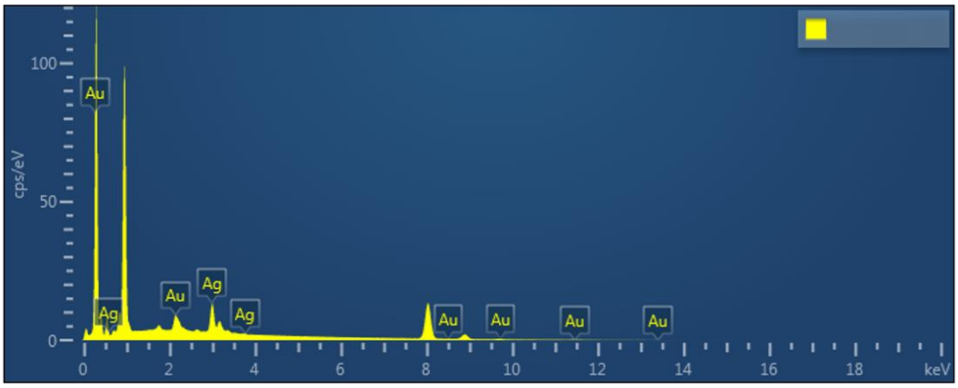

Figure S2. The element contents of Au and Ag within the SERS active area.

Table S1. The corresponding statistics from **Figure S1**.

Elements	Mass percent (%)	Atom percent (%)
Au	27.84	17.44
Ag	72,16	82.56

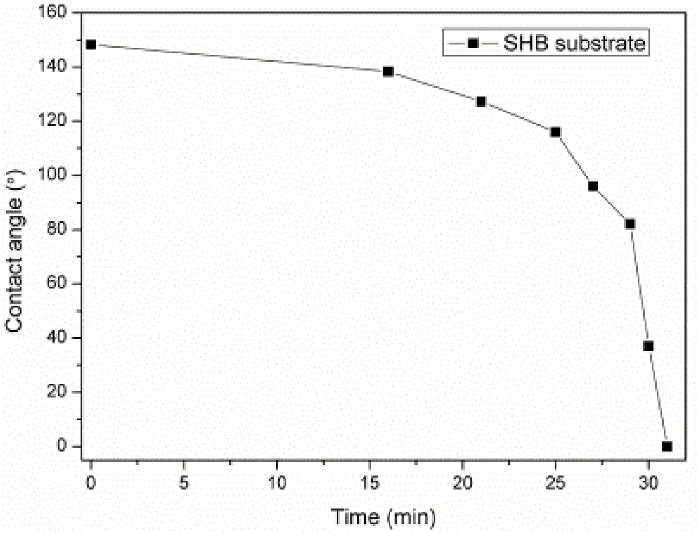

Figure S3. The dependence of contact angle on the evaporation time.

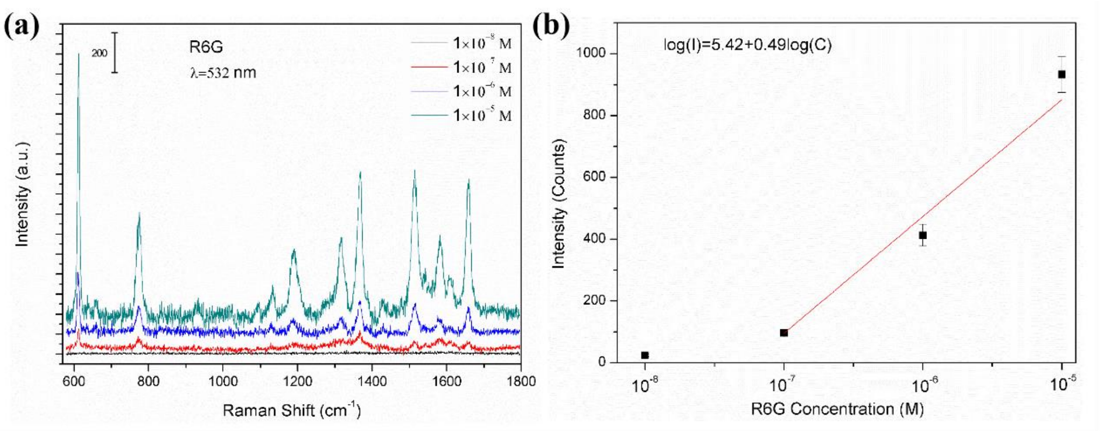

Figure S4. (a) SERS spectra of different concentrations (1×10^{-5} to 1×10^{-8} M) of R6G on normal SERS substrate with Au/Ag alloy-PAN, the detection limit of R6G on such substrate was measured to be 10^{-8} M. (b) the corresponding log-log plot of the average intensity of SERS signals at 612 cm^{-1} and R6G concentration.

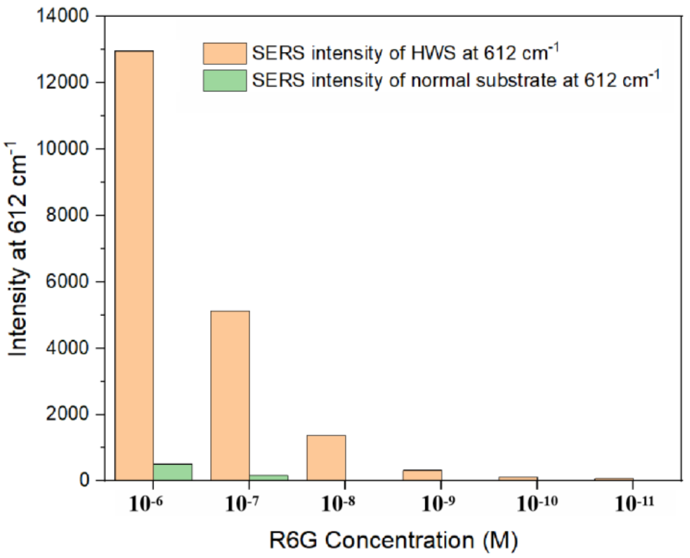

Figure S5. SERS intensity at 612 cm^{-1} of HWS (orange) and normal SERS substrate (green).

Enhanced Factor (EF) of the normal SERS substrate

The EF could be calculated by the following equation,

$$EF=(I_{SERS}/C_{SERS})/(I_0/C_0)$$

Where I_0 and C_0 represent the SERS signal intensities of R6G molecules on pure PAN nanofibers based substrate sample and corresponding R6G molecules concentration, respectively. In this case, the concentration of R6G molecules was 1×10^{-1} M (Figure S6). I_{SERS} and C_{SERS} represent the SERS signal intensities of R6G molecules on the normal SERS substrate with Au/Ag alloy-PAN and corresponding R6G molecules concentration, respectively. The concentration of R6G molecules

was 1 × 10^{-8} M. Therefore, the result calculated by the equation mentioned above was ~1.15 ×10^7 at 612 cm^{-1}.

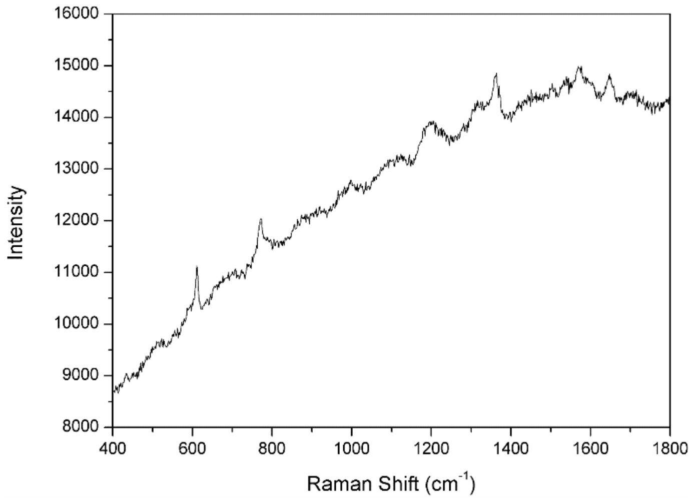

Figure S6. SERS signals of R6G molecules (1 × 10^{-1} M) on the pure PAN substrate.

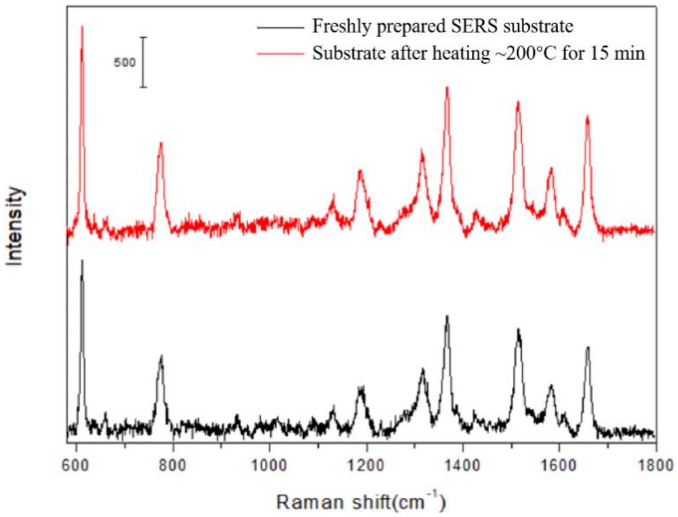

Figure S7. The evolution of SERS signals before and after heating process (200 °C for 15 min). In this respect, the results of both substrates showed that there were almost the same signal intensity, illustrating the thermal stability for SERS application.
